# Unexpected Responses of Bean Leaf Size to Elevated CO_2_

**DOI:** 10.3390/plants11070908

**Published:** 2022-03-29

**Authors:** James Bunce

**Affiliations:** Adaptive Cropping Systems Laboratory, USDA-ARS, Beltsville, MD 20705, USA; buncejames49@gmail.com; Tel.: +1-410-271-8177

**Keywords:** leaf size, leaf area, elevated CO_2_, temperature, diurnal, leaf water potential, turgor pressure, common bean, *Phaseolus vulgaris*

## Abstract

CO_2_ is currently a growth-limiting resource for plants with C_3_ metabolism, and elevated CO_2_ also often reduces stomatal conductance, reducing plant water stress. Increased photosynthesis and improved water status might be expected to result in increased leaf size. It is therefore unexpected that leaf size is in some cases reduced in plants grown at elevated CO_2_, and also unexpected that elevated CO_2_ applied only during darkness can increase leaf size. These experiments compared leaf size responses to day and/or night elevated CO_2_ in six cultivars of *Phaseolus vulgaris* grown with either constant or varying temperature in controlled environment chambers. Diverse responses of leaf size to elevated CO_2_ were found among the cultivars, including increased leaf size with elevated CO_2_ applied only during darkness in some cultivars and temperature regimes. However, leaf size responses to elevated CO_2_ and cultivar differences in response were unrelated to differences in leaf water potential or turgor pressure.

## 1. Introduction

The plant physiological processes affected by the concentration of CO_2_ in the air that are most directly related to plant production are photosynthesis and leaf area development. Much more research has been directed toward measuring, understanding, and modelling plant photosynthetic responses to rising atmospheric CO_2_ than toward CO_2_ effects on leaf area development. This is not because CO_2_ effects on leaf area are less important to plant growth stimulation at elevated CO_2_. For example, even in soybean, which has only minor downregulation of photosynthesis during growth at elevated CO_2_, and therefore has a large stimulation in photosynthesis per unit leaf area, increased leaf size at elevated CO_2_ was a large component of its overall growth stimulation [1].

Early work testing plant responses to elevated CO_2_ indicated that leaf size was often, but not always, increased by elevated CO_2_ (e.g., [2,3], and reviewed in [4]), and more recent work has also indicated increases, decreases, and no change in leaf size at elevated CO_2_ in a range of species. For example, Manderscheid et al. [5] reported increased leaf size at elevated CO_2_ in sugarbeet, as did Bunce [6] in common bean, and Song et al. in soybean [1]. On the other hand, Kim et al. [7] and Tsutsumi et al. [8] both found decreased leaf area or leaf size in rice at elevated CO_2_, as did Brinkoff in perennial ryegrass [9], and decreased leaf size was also found by McGranahan and Poling [10] in barley, wheat, maize, oats, sorghum, pinto bean, and sunflower. Yu and Korner [11] and Kizildeniz et al. [12] found no effect of elevated CO_2_ on leaf size in tomato and grape leaves, respectively. A recent meta-analysis of responses of C_3_ plants indicated an average slight linear increase in leaf size with increasing CO_2_ [13], but with relatively low consistency. Ultimately, leaf size is determined by the combination of cell numbers and cell size. Cell number per leaf is determined relatively early during leaf expansion. Cell expansion requires turgor pressure, but relationships between rates of expansion and turgor pressure vary with multiple endogenous and exogenous factors [3,4].

In a study of four herbaceous species, leaf extension rate was increased by elevated CO_2_ primarily at night [14], as also occurred in poplar [15]. In a study of two cultivars of common bean in the field, exposure to elevated CO_2_ only at night increased leaf size in one cultivar but not in the other [6]. Daytime only CO_2_ elevation did not increase leaf size in either cultivar, whereas continuous elevation CO_2_ did increase leaf size in both cultivars [6].

Partial stomatal closure is a frequent response to elevated CO_2_ treatments [16], and might result in reduced transpiration rates, and higher daytime leaf water potentials and turgor pressures. Higher turgor pressure might increase the expansion rates of developing leaves and the final leaf size. However, none of this would explain how elevated CO_2_ at night would affect leaf size. One possibility is that stomatal closure at night is incomplete [17], and that CO_2_ at night could affect stomatal conductance, leaf water potential, turgor pressure, leaf expansion rate, and final leaf size. Arguing against this scenario is the fact that leaf to air water vapor pressure differences at night in the field at Beltsville, Maryland, are usually very low, so that any differences in stomatal conductance between CO_2_ treatments would have very little impact on leaf water potential. This scenario would also fail to explain why CO_2_ elevation only during the daytime did not affect leaf size, or why the two common bean cultivars in a prior experiment differed in leaf size response to CO_2_ elevation at night.

The purpose of these experiments was first to learn whether the field responses of leaf size of the two cultivars of common bean to the CO_2_ treatments could be duplicated under more controlled conditions; secondly, to determine the leaf size responses of several other cultivars to day and/or night elevated CO_2_; and thirdly, to test for the involvement of leaf water potential and turgor pressure in CO_2_ treatment effects on leaf size.

## 2. Results

**Figure 1:**
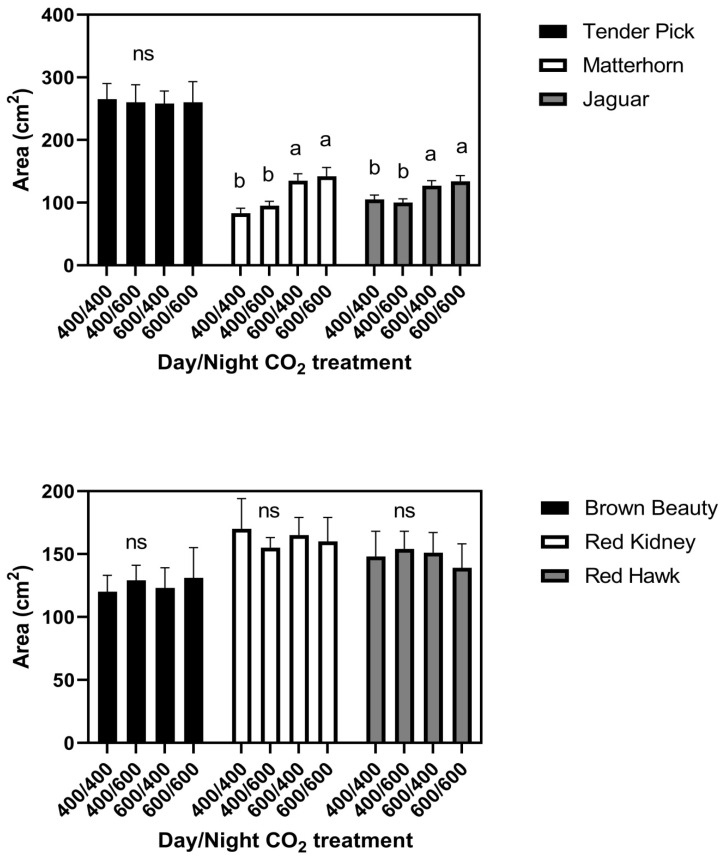
Area of terminal leaflets of third mainstem trifoliolate leaves of six cultivars of common bean grown with four day/night CO_2_ concentrations (μmol mol^−1^) at constant 23 °C. Bars indicate standard deviations, and different letters within cultivars indicate differences among CO_2_ treatments, using ANOVA at *p* = 0.05, and ns indicates no significant differences among CO_2_ treatments.

**Figure 2:**
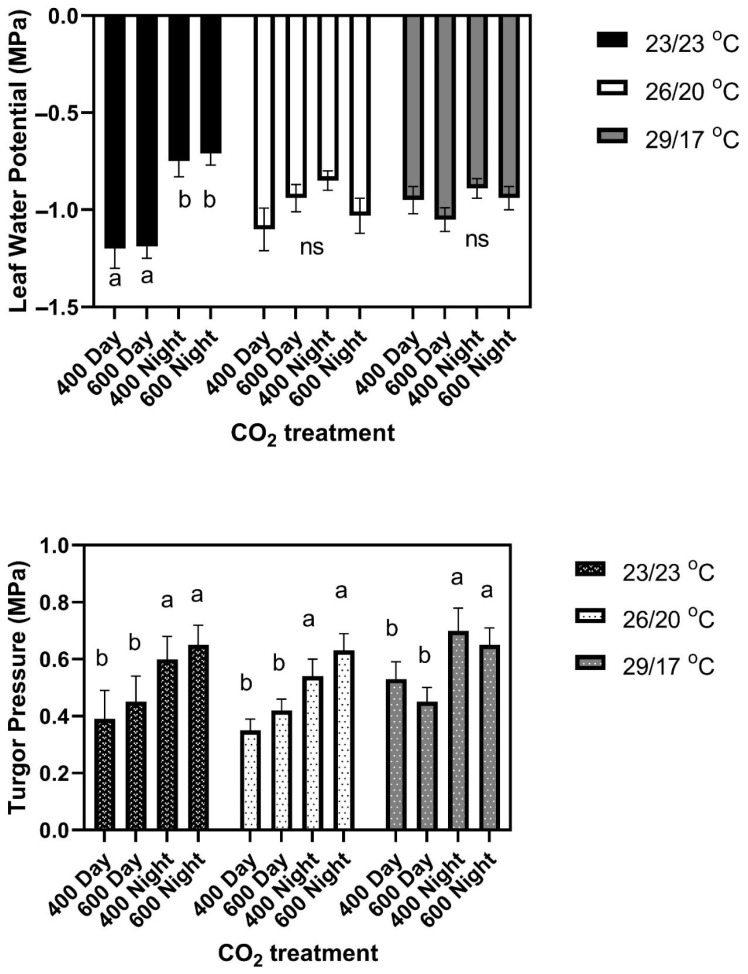
Leaf water potentials and turgor pressures of leaves of common bean sampled during the daytime or at night, with daytime or night growth CO_2_ concentrations of either 400 or 600 μmol mol^−1^, grown with three day/night temperature regimes. Values are the means for six cultivars, which did not differ significantly from each other. Bars indicate standard deviations, and different letters within treatments indicate differences among treatment means, using ANOVA at *p* = 0.05, and ns indicates no significant differences among CO_2_ treatments. Mean leaf water potentials and turgor pressures, and standard deviations for each cultivar and for all three temperature regimes are listed in Supplemental Table S1.

At the constant 23 °C growth condition, elevated CO_2_ given either continuously or only during the day increased leaf size in the cultivars Jaguar and Matterhorn (Figure 1). Elevated CO_2_ provided only at night did not affect leaf size in these two cultivars. None of the CO_2_ treatments significantly affected leaf size in any of the other four cultivars in this temperature regime (Figure 1). There were no differences among cultivars in leaf water potential or turgor pressure under any CO_2_ treatment conditions. Leaf water potentials averaged −1.20 ± 0.07 MPa in the daytime and −0.73 ± 0.05 MPa at night for all CO_2_ treatments and cultivars (Figure 2). Turgor pressures across cultivars averaged 0.45 MPa in the day time, and 0.60 Pa at night, with no significant differences among CO_2_ treatments (Figure 2). Mean leaf water potentials and turgor pressures, and standard deviations for each cultivar and for all three temperature regimes, are listed in Supplemental Table S1.

**Figure 3:**
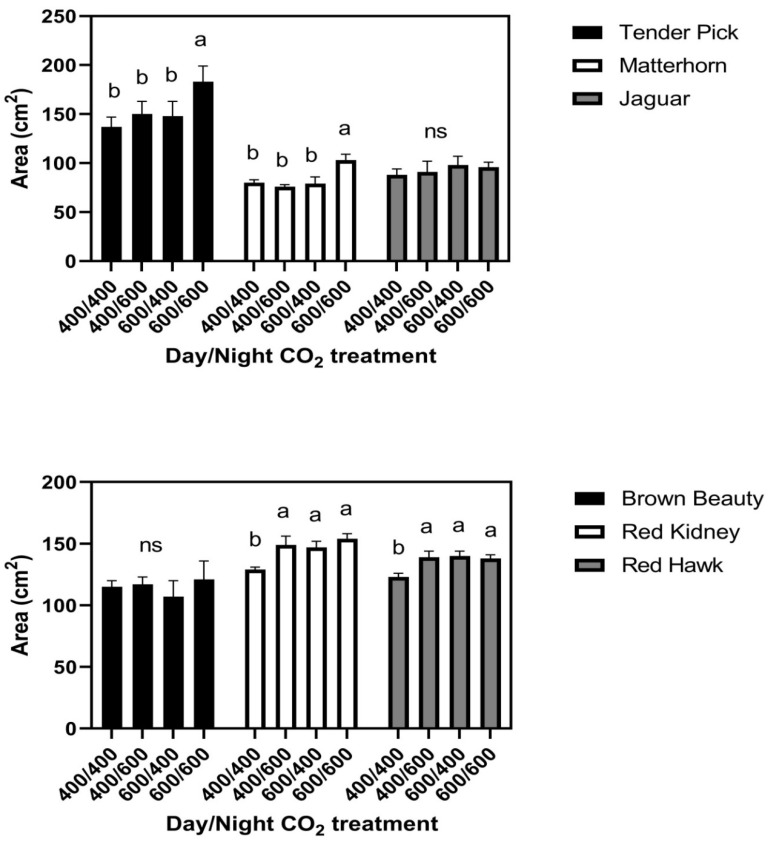
Area of terminal leaflets of third mainstem trifoliolate leaves of six cultivars of common bean grown with four day/night CO_2_ concentrations (μmol mol^−1^), with day/night temperatures of 26/20 °C. Bars indicate standard deviations, and different letters within cultivars indicate differences among CO_2_ treatments, using ANOVA at *p* = 0.05, and ns indicates no significant differences among CO_2_ treatments.

In the 26/20 °C day/night temperature regime, responses of leaf size to the day/night CO_2_ treatments fell into three groups, with two cultivars in each group. There were no CO_2_ treatment effects on leaf size in Brown Beauty or Jaguar (Figure 3). In Tenderpick and Matterhorn, elevated CO_2_ given continuously increased leaf size, but elevated CO_2_ only at night or only in the daytime had no effect on leaf size compared with constant ambient CO_2_ (Figure 3). In Red Hawk and Red Kidney, elevated CO_2_ during either the day or the night continuously increased leaf size (Figure 3). There were no significant differences among cultivars in leaf water potential either day or night, and no significant effects of CO_2_ treatment (Figure 2). Turgor pressure averaged higher at night than during the daytime in all cultivars (Figure 2).

**Figure 4:**
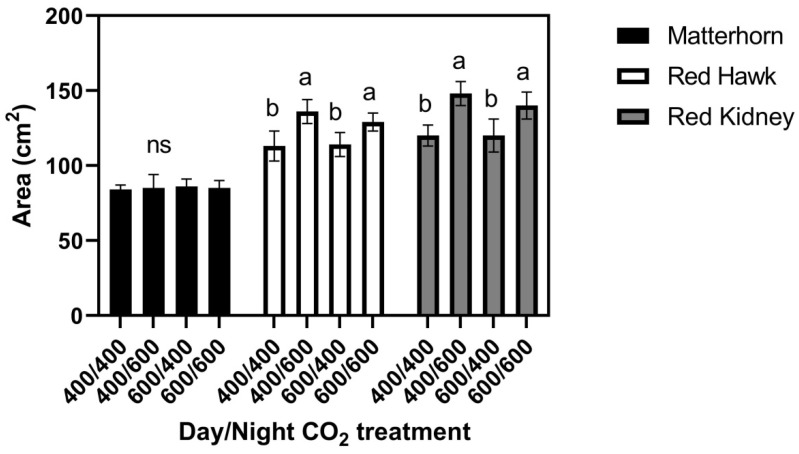
Area of terminal leaflets of third mainstem trifoliolate leaves of six cultivars of common bean grown with four day/night CO_2_ concentrations (μmol mol^−1^), with day/night temperatures of 29/17 °C. Bars indicate standard deviations, and different letters within cultivars indicate differences among CO_2_ treatments, using ANOVA at *p* = 0.05, and ns indicates no significant differences among CO_2_ treatments.

When three cultivars were grown at 29/17 °C, leaf size in Matterhorn did not differ among the four CO_2_ treatments (Figure 4), while both Red Hawk and Red Kidney had larger leaves when grown at 400/600 and 600/600 μmol mol^−1^ day/night CO_2_ concentrations than when grown at 600/400 or 400/400 μmol mol^−1^ Figure 4. For Red Hawk and Red Kidney, these leaf size patterns mimicked the response of Red Kidney previously observed in the field [6]. Leaf water potentials did not differ significantly between cultivars or CO_2_ treatments either during the light or the dark (Figure 2). Turgor pressures averaged higher at night than in the daytime (Figure 2), with no significant differences between cultivars or CO_2_ treatments.

## 3. Discussion

These indoor experiments were successful in duplicating the contrasting leaf size responses to day and/or night elevation of CO_2_ observed in field experiments in the cultivars Tenderpick and Red Kidney [6]. In the field, leaf size in Tenderpick was increased only by continuous elevation of CO_2_. In these experiments in controlled environment chambers, this response occurred in both the cultivars Tenderpick and Matterhorn when grown at 26/20 °C day/night temperatures, and in Matterhorn when grown at 29/17 °C. In the field experiment, leaf size in Red Kidney was increased to the same extent by elevated CO_2_ provided only at night, or both night and day, but not when provided only in the daytime. In these indoor experiments, that same leaf size response occurred in both Red Kidney and Red Hawk when grown at 29/17 °C day/night temperatures. The importance of the day/night temperature regime to the responses of leaf size to CO_2_ elevation is illustrated by the elimination of leaf size responses to elevated CO_2_ in Matterhorn as the amplitude of the day/night temperature difference increased, and the lack of CO_2_ effect on leaf size in Red Kidney and Red Hawk at constant temperature contrasting with strong responses with day/night temperature differences. These contrasting responses of the cultivars to the CO_2_ treatments occurred despite all cultivars being grown simultaneously in the same chamber.

Neither leaf water potentials nor turgor pressures, either in daytime or at night, helped to explain the cultivar differences in leaf size response to the temperature or CO_2_ treatment regimes, because no significant differences occurred among cultivars in either parameter in any environment. Leaf water potentials were significantly lower in daytime only in the constant temperature regime. Turgor pressures averaged about 0.2 MPa higher at night than in daytime in all temperature regimes, perhaps being a more sensitive indicator of water status than leaf water potential. Ferris and Taylor [14] and Gardner et al. [3] were also unable to relate CO_2_ effects on leaf extension rates to treatment differences in turgor or leaf water potential. All of these results suggest some sort of metabolic control of leaf expansion rather than control by transpiration or leaf water potential [15]. Seneweera and Conroy [18] concluded that faster expansion of wheat leaves at elevated CO_2_ was related to greater availability of soluble carbohydrates for export from mature to developing leaves, but how this might relate to day/night patterns of leaf expansion or effects of CO_2_ at night on expansion are unclear.

There are certain similarities between leaf size responses to CO_2_ and to the light environment, with increases in light sometimes increasing and sometimes decreasing leaf size, depending on the light level and the species (e.g., [19,20]). For responses of expansion to light regimes, changes in gene expression, and changes in auxin, gibberellin, and cytokinin and phototropin content have sometimes been identified as controlling factors (e.g., [21,22,23]), but to our knowledge there is no similar data for responses of leaf size to CO_2_. In previous work with bean primary leaves, elevated CO_2_ applied throughout leaf development did not affect final leaf size, but it increased final size when applied only during the cell expansion phase [24], which suggests that it decreased cell proliferation. In ryegrass leaves increasing CO_2_ concentration increased leaf elongation rate during the daytime, but decreased it during the night [25], with the 24-h rates unaffected.

In some annual crops, such as determinate cultivars of common beans, canopies often have a fairly low leaf area index for much of the yield formation period. In this situation, differences in leaf size among CO_2_ treatments may have a large impact on seed yield, which makes cultivar differences in CO_2_ effects on leaf size an important factor in yield responses. For example, the relative seed yield increase was nearly the same in Tenderpick and Red Kidney when elevated CO_2_ was applied only in the daytime, but was about 20% larger in Red Kidney than in Tenderpick when CO_2_ was elevated continuously [6]. Clearly, elevating CO_2_ only during the daytime, as many free air CO_2_ enrichment systems do, would misrepresent some cultivar differences in yield responses to rising atmospheric CO_2_ in common bean, and probably in other species as well, simply because of the complex responses of leaf size to elevated CO_2_. Effects of CO_2_ on plants during darkness have been recognized for several years (e.g., [26,27]). In the context of climate change, only free-air CO_2_ enrichment (FACE) experiments have frequently chosen not to increase CO_2_ at night as well as daytime, sometimes because of the expense, and sometimes because of a lack of wind to distribute the CO_2_ across the plot. The latter problem can be overcome by using area-distributed FACE systems [28]. Soybean yield at elevated CO_2_ in FACE was larger when elevated CO_2_ was applied for 24 h per day than when applied only in the daytime [29], as reported for beans in open top chambers [6]. Responses of leaf size to CO_2_ both day and night, and interactions with temperature remain unexplained plant physiological phenomena, but have important practical implications for efforts to adapt crops to the rising atmospheric CO_2_.

## 4. Materials and Methods

Six cultivars of common bean (*Phaseolus vulgaris* L.), namely, Brown Beauty, Red Kidney, Jaguar, Matterhorn, Red Hawk, and Tenderpick, were grown in indoor controlled environment chambers. Tenderpick and Red Kidney were used, because this was an attempt to duplicate and understand the leaf size responses of these two cultivars observed previously under field conditions. The other four cultivars were selected as also being determinate, bush-type bean plants, as are Tenderpick and Red Kidney. Four chambers, each with 3.7 m^2^ ground area, were utilized, with day/night CO_2_ concentrations controlled to 400/400, 400/600, 600/400, or 600/600 μmol mol^−1^. Light was provided for 12 h per day from a mixture of high-pressure sodium and metal halide lamps with dimmable ballasts, programmed to maintain a PPFD of 1000 μmol m^−2^ s^−1^ measured at the tops of the plants. Pure CO_2_, or CO_2_-free air was added to each chamber under the control of an absolute infrared CO_2_ analyzer (WMA-4 or WMA-5, PP Systems, Amesbury, MA, USA) whose output was sent to a proportional–integral–derivative (PID) controller. Peak to trough variation in CO_2_, as measured with an open path CO_2_ analyzer sampling at 0.1 Hz, was 15 μmol mol^−1^ at 400 μmol mol^−1^, and 22 μmol mol^−1^ at 600 μmol mol^−1^ control concentrations. Experiments using all cultivars were run with day/night air temperatures of 23/23 and 26/20 °C. Experiments with three cultivars, namely, Red Kidney, Matterhorn, and Red Hawk, were also run at 29/17 °C, because, for Red Kidney, the field responses of leaf size of were not precisely mimicked in either of the other temperature regimes, as they were for Tenderpick, and Red Hawk had responses similar to Red Kidney in the other environments tested here. These temperature regimes were chosen to approximate growing season temperatures in Beltsville MD, where the mean is 23 °C, the average day and night temperatures are 26 and 20 °C, and the average daily maximum and minimum temperatures are 29 and 17 °C. In all experiments, temperature, humidity, CO_2_, and PPFD were logged every 2.5 min to a computer. Temperature was controlled to ±0.3 °C. Air saturation deficits for water vapor during the day time averaged 1.1, 1.3, and 1.6 kPa at 23, 26, and 29 °C air temperatures. Air saturation deficits at night were 0.4 to 0.6 kPa in all thermal regimes. Each combination of CO_2_ and temperature had three chamber replicates over time, with the four CO_2_ treatments rotated among chambers.

Plants were grown rooted in a medium grade of vermiculite and watered daily with a complete nutrient solution containing 14.5 mM nitrogen. Plants were grown in plastic bins, 0.30 m^2^ in area, and 50 cm in depth, with one bin per cultivar in each chamber. Seeds were overplanted, and seedlings thinned for uniformity of emergence time and for even spacing to 10 plants per bin. The position of the bin for each cultivar within the chambers was kept the same for all four chambers within a replicate experiment, and randomized between replicate experiments.

Plants were grown until the third mainstem trifoliolate leaf was fully expanded, and then the areas of the terminal leaflets of the third trifoliolate leaves of all plants were measured with a leaf area meter (LI-3000C, LiCor Inc., Lincoln, NB, USA). Prior to the final harvest, when third trifoliolate leaves were less than half of their final area, leaf water potentials and turgor pressures were measured both in the dark and in the light. Leaf discs were removed from leaves either shortly before lights came on, or at least two hours after lights came on. Leaf water potentials were measured on the excised discs using an HR33 dew point hygrometer and C-52 sample chambers (Wescor Inc., Logan, UT, USA). The leaf discs were then sealed in the sample cups, frozen in a −80 °C freezer, thawed, and water potential measured again, to indicate osmotic potential. Turgor pressure was calculated as the difference between the leaf total and osmotic water potentials. These estimates of turgor are subject to some error caused by dilution of intracellular water by less concentrated water in cell walls, upon thawing. For each chamber run, for each cultivar, two replicate leaf discs from two different randomly selected plants were collected in the dark and in the light.

Leaf size responses of the cultivars to the CO_2_ and temperature treatments were compared by analysis of variance, initially using three-way ANOVA to test for differences in response to cultivar, CO_2_, and temperature, and their interactions. Because responses of size to cultivar and CO_2_ interacted with temperature, each temperature was then analyzed separately using two-way ANOVA. For each temperature, the interaction of cultivar and CO_2_ was significant. These two-way ANOVAs are presented in Appendix A and Table A1, Table A2 and Table A3. Because for each temperature, the cultivar by CO_2_ interaction was significant, the response of each cultivar to the CO_2_ treatments is shown in Figure 1, Figure 2 and Figure 4. For daytime and night time leaf water potential and turgor pressure, the same 3-way analyses of variance indicated no significant effects of cultivar or interactions of cultivar with CO_2_ or temperature, and therefore two-way ANOVA was used (Table A4 and Table A5) to test for effects of CO_2_ and temperature across all cultivars.

## Data Availability

The data presented in this study are available on request from the author.

## Supplementary Materials

The following supporting information can be downloaded at: https://www.mdpi.com/article/10.3390/plants11070908/s1, Table S1: Lists of mean leaf water potentials and turgor pressures, and standard deviations for each cul-tivar and for all three temperature regimes.

## Appendix A

**Table A1:** Analysis of variance for area of third leaves of six bean cultivars grown at 23 °C with four day/night CO_2_ conditions.

Source	Degrees of Freedom	Sum of Squares	Mean Square	F-Value	*p*-Value
CO_2_	3	2498	833	2.81	0.0493
Cultivar	5	183,917	36,783	124.1	0.0001
CO_2_ × Cultivar	15	8674	578	1.951	0.0408
Residual	48	14,226	296		

**Table A2:** Analysis of variance for area of third leaves of six bean cultivars grown at 26/20 °C with four day/night CO_2_ conditions.

Source	Degrees of Freedom	Sum of Squares	Mean Square	F-Value	*p*-Value
CO_2_	3	3869	1290	18.0	0.0001
Cultivar	5	48,246	9649	134.7	0.0001
CO_2_ × Cultivar	15	3222	215	2.998	0.0020
Residual	48	3438	71.6		

**Table A3:** Analysis of variance for area of third leaves of three bean cultivars grown at 29/17 °C with four day/night CO_2_ conditions.

Source	Degrees of Freedom	Sum of Squares	Mean Square	F-Value	*p*-Value
CO_2_	3	1966	665	10.94	0.0001
Cultivar	2	14,936	7468	124.6	0.0001
CO_2_ × Cultivar	6	1022	170	2.843	0.0310
Residual	24	1438	59.9		

**Table A4:** Analysis of variance for leaf water potential of bean leaves under four day/night CO_2_ conditions, at three growth temperatures.

Source	Degrees of Freedom	Sum of Squares	Mean Square	F-Value	*p*-Value
Day/night CO_2_	3	0.4174	0.1391	25.37	0.0001
Temperature	2	0.00335	0.00168	0.3055	0.7396
CO_2_ × T	6	0.3805	0.06341	11.56	0.0001
Residual	24	0.1316	0.00548		

**Table A5:** Analysis of variance for turgor pressure of bean leaves under four day/night CO_2_ conditions, at three growth temperatures.

Source	Degrees of Freedom	Sum of Squares	Mean Square	F-Value	*p*-Value
Day/night CO_2_	3	0.3534	0.1178	25.29	0.0001
Temperature	2	0.0581	0.0290	6.231	0.0066
CO_2_ × T	6	0.0374	0.00623	1.336	0.2799
Residual	24	0.1118	0.00466		
